# Anther Culture Efficiency in Quality Hybrid Rice: A Comparison between Hybrid Rice and Its Ratooned Plants

**DOI:** 10.3390/plants9101306

**Published:** 2020-10-02

**Authors:** Snigdha Samir Pattnaik, Byomkesh Dash, Sudhansu Sekhar Bhuyan, Jawahar Lal Katara, C. Parameswaran, Ramlakhan Verma, Narayanaperumal Ramesh, Sanghamitra Samantaray

**Affiliations:** 1 Crop Improvement Division (CID), ICAR-National Rice Research Institute, Cuttack 753006, Odisha, India; snigdhasameer89@gmail.com (S.S.P.); dashbyom.k@gmail.com (B.D.); sb.biotechnology@gmail.com (S.S.B.); jawaharbt@gmail.com (J.L.K.); agriparames07@gmail.com (C.P.); ram.pantvarsity@gmail.com (R.V.); 2 Department of Biotechnology, J.J. College of Arts and Sciences, affiliated to Bharathidasan University, Pudukkottai 622422, Tamilnadu, India; nprg@rediffmail.com

**Keywords:** androgenesis, doubled haploid, hybrid rice, ratoon, regeneration, spike

## Abstract

An immense increase in human population along with diminished lands necessitates the increase of rice production since, it serves the human population as a staple food. Though rice hybrids (RH) are showing considerable yield enhancement over inbreds in terms of both quality and quantity, farmers’ adoption of hybrid rice technology has been much slower than expected because of several constraints such as seed cost and quality. Doubled haploid (DH) technology was considered useful for the development of inbred lines from rice hybrids in a single generation. Androgenesis shows its significance in development of DHs in rice which requires an efficient method to establish the production of large population. To start the anther culture, anthers are the main component of androgenesis to be isolated from unopened spikes. However, the duration of spikes availability for anther culture coupled with the segregation of rice hybrids in the next generation requires the main crop be ratooned to reduce the cost of cultivation. Therefore, the efficiency of the androgenic method was tested in main crop using a quality *indica* rice hybrid, 27P63 and its ratooned ones. The effects of various factors such as cold temperature pre-treatment of boots, treatment duration, and different combination of plant growth regulators (PGR) on callus response along with shoot regeneration were tested for development of DHs from both ratooned and non-ratooned plants. The N6 medium supplemented with 2.0 mg/L 2,4-D (2,4-dichlrophenoxy acetic acid), 0.5 mg/L BAP (6-benzylamino purine), and 30 g/L maltose was found to be most effective for callusing as compared to MS (Murashige and Skoog) medium. The N6 media inducted calli showed maximum response rate for green shoot regeneration in MS media supplemented with 0.5 mg/L NAA (1-napthaleneacetic acid), 0.5 mg/L Kn (Kinetin; 6-furfurylaminopurine), 1.5 mg/L BAP and 30 g/L sucrose after 2 weeks of culture. The pre-treatment of spikes at 10 °C for 2 d followed by a 7th and 8th d were found to be most effective for callusing as well as for regeneration, producing a total of 343 green plants from ratooned and main rice hybrid, 27P63. Morpho-agronomic trait-based assessment of ploidy status revealed 94.46% diploids, 3.49% polyploids, 0.58% mixploids, and 1.45% haploids. Microsatellite markers could authenticate all 324 fertile diploids as true DHs. Though this study shows a reduction in generation of DHs from ratooned plants as compared to the main crop, manipulation of chemical factors could optimize the method to enhance the production of considerable number of DHs. Utilization of ratooned of hybrid rice in androgenesis would save time and cost of cultivation.

## 1. Introduction

Rice (*Oryza sativa* L.) is the world’s most important food crop, cultivated in Southeast Asian region which fulfils the demand of the almost half of the population in the world [1]. Though the production of rice has increased almost three fold over the last decades [2], the continued increase in sustaining yield for development of high-yielding varieties is needed to meet the increasing demand of global population. Rice hybrids have shown significant yield advantages of more than 30% over in-bred varieties [3]. However, farmers’ adoption of hybrid rice technology has been much slower than expected because of several constraints such as seed cost and quality. Additionally, the production of RH requires a three lines system (A, B, and R lines) which is more tedious and requires skilled personnel. These constraints in hybrid rice attract attention to adopt DH technology as there is a possibility to produce filial 1 (F1)-performing high yielding DH lines from the heterotic F1s of rice by fixing hybrid performance exerting the partial to complete dominance in homozygous lines [4,5].

There are a number of methods available to generate DHs out of which in vitro methods were found to be more suitable for its production. Out of two in vitro methods i.e., gynogenesis and androgenesis, anther culture shows its effectiveness and applicability in the production of homogenous pure lines in rice [2,5]. Conversely, the development of pure lines or inbred lines by conventional methods takes 6–8 years. Though anther culture has shown its significance in improvement of *japonica* rice [6], *indica* rice is found to be recalcitrant to anther culture [7]. Even though a number of studies on anther culture have been carried out in *indica* rice hybrids [4,5], still it requires an intensive study to clear the bottle necks in optimization of method for androgenesis.

Usually, rice grows in two seasons i.e., rabi and kharif, particularly in eastern India. Standardization of androgenesis method requires anthers as explants from unopened spikes; obtaining such explants takes a maximum period of one month which is sometimes insufficient for establishing an efficient anther culture method. Besides, the spikes containing the anthers cannot be used as the F_1_s of hybrid rice, as it segregates in the next generation. Therefore, culturing a large number of anthers in the proper stages requires the ratooned of hybrid rice as the ratooned crop has shown the advantages such as reduced cost of production through saving in land preparation and care of the plant. To date, there has been only a single report available on the response of androgenesis in main crop and the ratooned ones in *japonica* rice [8]. As *indica* rice is predominantly grown in more than 70% area in world, particularly in Southeast Asian countries [8,9], the present investigation was undertaken to study the efficiency of anther culture in generation of DHs from an *indica* quality hybrid rice, 27P63, and its ratooned plants.

## 2. Results

### 2.1. Indicator for Microspore Stage

Early to late-uninucleate stage of microspores were more responsive during androgenesis as compared to other stages. As determining the stages of anther (microspore) using acetocarmine stain is not possible every time, a morphological indicator i.e., distance between the flag leaf and the first leaf of rice plant boot was used to determine the stage. It was observed that the boots having 15–18 cm distance between their flag and first leaf carry the most numbers of microspores in the early to late uni-nucleate stage; the explants were chosen accordingly for androgenesis. The ratoons developed from the cut stalk of the main plants by 25 cm above ground level showed diminutive growth leading to reduction in the distance between the first leaf and flag leaf. Post cytological studies of the ratooned boots determined the correct stage of anthers which were correlated to the average distance of 5–10 cm between flag leaf and the first leaf.

### 2.2. Callus Induction

**Figure 1:**
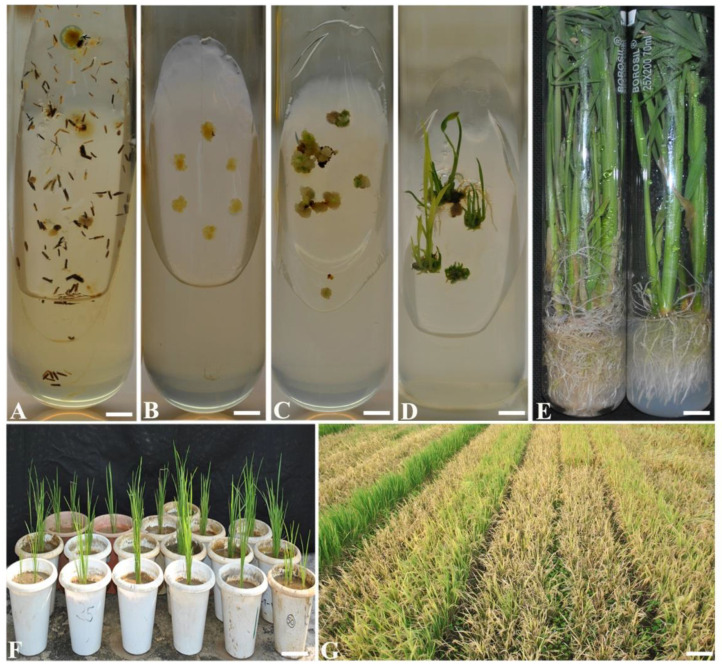
Development of regenerants from non-ratooned and ratooned of 27P63 through androgenesis. (**A**) Calli induction from microspores of anthers (bar 2 mm). (**B**) Calli on regeneration media for green shoot regeneration (bar 2 mm). (**C**) Callus with green spots (bar 2 mm). (**D**) Well grown green shoot regeneration from calli (bar 2 mm). (**E**) Rooting in microshoots (bar 5 mm). (**F**) Acclimatization of anther derived plants (bar 15 cm). (**G**) Variability in DH lines grown in field (bar 20 cm).

Anthers containing the suitable stage of microspores when cultured in MS and N6 media changed the color from light yellow to dark-brown and swelled in size after 5–8 d of culture. Thereafter, the wall of anthers cracked with a small globular protuberance which gradually turned into a mass of cell after 2–3 weeks (Figure 1A). The anthers derived from non-ratooned rice hybrid performed better in terms of callus induction than ratooned plants in all treatments (Table 1). Differential responses in calli induction were observed among the six media used both in ratooned and non-ratooned derived anthers. Variability in size, number, and color of calli were observed for both the anther sources used. Anthers derived from non-ratooned plants responded for higher callusing (21.66 ± 0.75%) with light yellowish and compact texture in all the treatments, while anthers from ratooned plants showed 11.66 ± 0.30% callusing; a number of calli exhibited friable structure. The over-all calli frequency varied from 21.66 ± 0.75 to 0.36 ± 0.26% in N6, whereas 13.66 ± 0.05 to 0.10 ± 0.10% in MS for both type of plants. The highest frequency of anthers producing multiple calli was observed in N6 based N1 media supplemented with 2.0 mg/L 2,4-D and 0.5 mg/L BAP in both non-ratooned and ratooned plants.

### 2.3. Shoot Regeneration

The calli emerged from the cultured anthers of both ratooned and non-ratooned plants were transferred at the size of 1–2 mm to two different regeneration media (Figure 1B). Within two weeks of culture, the transferred calli started differentiating into lump of callus with green spots and gradually turned into green shoots (Figure 1C). Further, the shoots were found elongated (Figure 1D). It was observed that the 2-d pre-incubated calli grown in N1 media of non-ratooned plants showed significantly higher green shoot regeneration followed by the 7th d and 8th d pretreatments at 10 °C. However, highest green plant regeneration (76.00 ± 0.30%) was observed in MS1 {MS + BAP (1.5 mg/L) + Kn (0.5 mg/L) + NAA (0.5 mg/L) + sucrose (30 g/L)} from the calli induced in N1 media (Table 2). The overall study of both ratooned and non-ratooned suggested that the N6 derived callus were more effective for green shoot regeneration. Between the two-regeneration media (MS1 and MS2) tested for shoot regeneration, MS1 media was found to be most effective for green shoot regeneration. Regeneration percentage of non-ratooned derived calli developed in N6 media, varied from 76.00 ± 0.30 to 13.33 ± 0.30% and 27.66 ± 0.15 to 11.66 ± 0.25% in MS1 and MS2 media, respectively (Table 2). Differential response in regeneration of ratooned plants derived calli varied from 38.86 ± 0.26 to 10.63 ± 0.30% in MS1 and 19.96 ± 0.25 to 11.30 ± 0.37% in MS2. In general, non-ratooned-derived calli showed better shoot regeneration frequency than the ratooned ones.

### 2.4. Rooting and Acclimatization

The green shoot regenerants transferred to MS media supplemented with NAA (2.0 mg/L) + Kn (0.5 mg/L) + sucrose (50 g/L) induced 100% roots in both non-ratooned and ratooned plants (Figure 1E). Well-developed plants with proper root system were acclimatized and transferred, phase by phase to the net house (Figure 1F). Developed plantlets showed variability in terms of height, panicle length and grain types (Figure 1G).

### 2.5. Ploidy Evaluation

**Figure 2:**
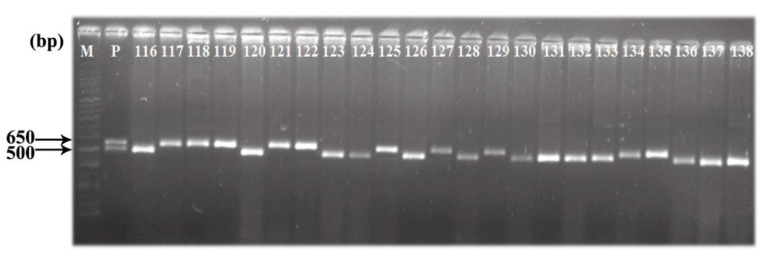
Identification of true DHs using STMS marker (RM23310). M: 100bp ladder taken as reference; P: Heterozygote rice hybrid 27P63; 116-138: Screened DHs.

Ploidy levels of the anther derived plants of both the ratooned and main crop were confirmed by assessing the morphological characters. In case of main crop, 207 regenerants showed normal morphological appearance with 80–95% grain fertility along with 3 polyploids and 3 haploids. Similarly, 117 regenerants developed from ratooned plants had 75–90% grain fertility with normal morphological appearance; 9, 2 and 2 plants were polyploids, haploids, and mixploids, respectively. Since, it is difficult to distinguish DHs from diploids through morphological characters, STMS markers were used to authenticate the true DHs. Interestingly, there was not a single heterozygote observed in the DH population; all 324 were observed to be 100% true DHs (Figure 2). Overall, 94.46% (324) DHs were achieved out of 343 green plants of which 207 DHs were derived from non-ratooned and 117 DHs were of ratooned plants.

## 3. Discussion

Cultivation of rice from sowing to maturity is generally cost intensive in terms of labor and other field-related inputs. Since, fresh spikes are required for production of DHs, it is required to grow the rice plants every season which is a costly affair. To make the DH production cost effective, ratooned crop was used to study its capacity for production of DHs which has not been exploited in tissue culture [8]. Even though there is variation in androgenic response within *indica* subspecies of rice [10], a comparison in production of DHs was made between ratooned and non-ratooned ones.

Well established anther culture often responds to *indica* rice genotypes with poor regeneration ability and increased albinism [11]. A number of physiological factors such as genotype, maturity of the donor plant, and microspore developmental stages play significant roles in governing the rice androgenesis. Besides, panicle pre-treatment, temperature and duration of pre-treatment, culture media, and growth conditions are some of the physical and chemical factors which cannot be ignored as they show their significance in establishment of efficient androgenic method for development of a considerable amount of DHs [5].

Among all the factors responsible for establishing efficient androgenic method, temperature and pre-treatment duration play a vital role in production of large amount of DHs. Our result showed that 2-d spike pretreatment at 10 °C produced maximum calli response in F1s of 27P63 at the mid-uninucleate stage followed by 7-d which supports the findings in *indica* rice hybrid [4]. Besides the pretreatment temperature and period, the PGRs play an important role in response of callusing. Our results showed that highest callusing was achieved in N6 media fortified with 2 mg/L 2,4-D, 0.5 mg/L BAP and 3% maltose as compared to other two media tested which contains 1.5–2.5 mg/L 2,4-D, 0.5–1.0 mg/L BAP, 0.1 mg/L Kn. Addition of 2 mg/L 2,4-D alone in the media was found quite efficient in production of reasonable rate of callus response in *indica*-*indica* crosses [12]. However, combinations of low concentration, 0.5 mg/L BAP with 2.0 mg/L 2,4-D enhances the callus induction frequency in our study particularly for the main crop which might be due to the synergistic effect of auxin and cytokinin concentration for callus response. This study shows the reduction in callus response of anthers up to 3.3 times in ratooned plants as compared to the main plants. Lower frequency of callusing along with friable texture was observed in the ratooned derived anthers as the same concentration of 2,4-D might not be appropriate for production of similar frequency of callus as the main crop.

In the present study, the shoot regeneration frequency was maximum in MS media supplemented with 1.5 mg/L BAP, 0.5 mg/L Kn and 0.5 mg/L NAA. However, minimum increase in the concentration of BAP and Kn significantly reduced the shoot regeneration frequency almost 3 times in main crop and 2 times in ratooned plants. It might be due the inadequate concentrations of PGRs used for balancing the endogenous level of auxin/cytokinin. The rice hybrid 27P63 exhibited the best green shoot regeneration as compared to other *indica* rice hybrids BS6444G (68.20%) and CRHR32 (71.66%) [4,5] of 7th and 2nd d pre-incubated boots, respectively. Surprisingly, there were no albinos observed in the regenerants both from ratooned and non-ratooned plants suggesting the alleged role of the spikes pre-incubation duration and temperature which prevented the albino regeneration from the calli. Prolonged pre-incubation condition often creates single base pair mutation from C to T leading to a missense mutation (Thr to Ile) causing albinism which is currently the most infuriating problem in androgenesis particularly in *indica* rice [13].

Assessment of ploidy level in the regenerants is the most important key step for application of androgenesis in the breeding program. Therefore, an accurate and dependable method for ploidy determination should be chosen judiciously keeping time- and cost-effectiveness in consideration. In this study, morphological assessment was found quite reliable in distinguishing diploids from other ploids which is rapid and easy to perform [14]. Though flow cytometry is an attractive approach to assess the ploidy levels of regenerants, its application is still limited in many labs due to high price of the equipment and higher cost per each analysis [15]. Besides, this method cannot discriminate DHs from diploids distinguishing heterzygotes of in vitro-raised plants. Employment of STMS markers could efficiently provide the information of allelic distribution of all 324 fertile diploids without any heterozygotes, authenticating the origin of all regenerants from the microspore and not from the somatic tissue of the F1s; all the 324 diploids are true DHs.

The protocol was established by manipulating PGRs which produce reasonable callusing and shoot regeneration in *indica* rice hybrid, but it has its limitations in the production of green plants in ratooned crops. However, the reduction of androgenesis in ratooned plants can be restored equivalent to normal/non-ratooned plants with minor manipulation of PGRs, along with reasonable changes in the carbon or nitrogen sources to balance the deficiency of carbohydrate and hormonal levels of the explant and growth conditions of ratooned plant. The friable calli texture of the ratooned plant opens up an idea for modification of 2,4-D concentration in the established media, achieving considerable callus response and subsequent shoot regeneration frequency. This study showed that minute changes in established media could lead to the development of an efficient method for androgenesis within *indica* subspecies as well as its ratooned counterparts.

The development of RIL (Recombinant inbred lines) for mapping any QTL (Quantitative Trait Loci)/gene and the production of inbred lines from F_1_s of any cross /hybrid rice through traditional breeding programs usually takes minimum of 6–8 years that to 99.22% homozygosity whereas DHs can achieve the 100% homozygosity within one year of time period. As only the F_1_ spikes carrying the anthers are utilized in production of DHs via androgenesis, achieving large population through androgenesis is not always feasible in a single season. In case of rice hybrids, F_1_s usually segregates in subsequent generation; reinitiating the F_1_ development is cost intensive. Moreover, the ratooned plants could be utilized in developing efficient androgenic protocol without any extra financial burden. Hence, there is a need to develop a systematic androgenic protocol for effective regeneration of DHs in ratooned plants which will definitely reduce the overall cost of re-cultivation of the main crop. Though a significant reduction was observed in achieving DHs from ratooned plants as compared to the main crop, ratooned could be cost effective and time saving in developing new F_1_s of the targeted hybrid if an efficient androgenic protocol was established. This study could open up new idea in generation of DHs using ratooned crops which not only save time but also reduce the financial costs.

## 4. Materials and Methods

### 4.1. Plant Material

**Figure 3:**
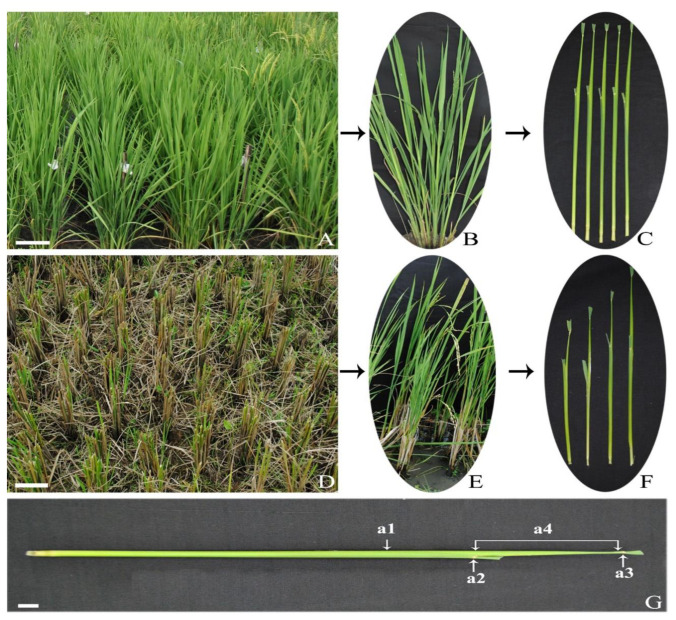
Explants sources. (**A**) Non-ratooned plants (bar 15 cm). (**B**) Close view of a single parent plant bearing fertile tillers. (**C**) Boots collected from the main crops. (**D**) Ratooned plants in field (bar 15 cm). (**E**) Close view of ratooned crop bearing boots. (**F**) Boots collected from the ratooned crops. (**G**) A panicle boot measured for the internode distance: (a1) leaf sheath-enclosed panicle, (a2) node of the penultimate leaf, (a3) node of the flag leaf, (a4) internode distance (bar 2 cm).

The rice hybrid, 27P63, developed by M/S Dupont Pioneer, Hyderabad, was used as the donor for anther culture, as it fetches an exuberant price in the market for the grain quality. It is a medium-height plant (100 cm) with erect non-lodging plant type and matures in 130–135 d. Later, post-harvesting these plants were ratooned i.e., cut 25 cm above ground part for re-tillering. Thirty-day-old seedlings were transplanted in a puddled field with precise spacing of 20 cm × 15 cm in kharif, 2017 after raising in nursery beds. Three split doses of nitrogen (N), phosphorus (P), and potassium (K) (100:50:50) were applied and necessary protection measures were taken. After the first successive harvesting of the main crops in kharif, 2017, these plants were ratooned in rabi, 2018 as the best harvesting time for good ratooning was when the culms were still greenish. The first phase panicles (non-ratooned) were collected at booting stage between 05:30–7:00am; the distance between the flag-leaf and subtending leaf was around 15–20 cm (Figure 3G) and microspores present in anther at the central (mid) part of the panicle were at the mid-to-late uninucleate stage. Followed the first harvest of the panicles, the boots re-sprouting from the base of the plant were considered as the ratoons (Figure 3D,E). Since flowering was found to be asynchronous, panicles of ratooned plants were harvested at the length of the booting distance around 5–14 cm (Figure 3F). After collection, both ratooned and non-ratooned panicles were wrapped in a moist (with water) cotton towel and kept in dark at 10 °C for 2–8-d as cold pre-treatment. To ensure the proper stage of microspore, anthers were fixed in acetic acid: ethanol in ratio (1:3) with 2% FeCl_3_ for 24 h followed by staining with 2% acetocarmine to trace out the stages of microspores in the anthers under microscope.

### 4.2. Anther Culture

The pre-treated boots were surface-sterilized with 70% ethyl alcohol for 5 min followed by 4% NaOCl (Merck, India) for 2–3 min and washed three times with sterilized distilled water. Anthers were removed from spikes under sterile conditions and tapped onto test tubes containing different media. For callus induction, two different nutrient media, MS [16] and N6 [17] were prepared with three different combinations and concentrations of BAP (0.5–1 mg/L), Kn (0.1 mg/L), and 2,4-D (1.5–2.5 mg/L) along with maltose (30 g/L) (Table 3). Prepared media was adjusted to pH 5.7–5.8 using 0.1N NaOH/0.1N HCl followed by solidification with 0.8% agar-agar (HiMedia, India) prior to autoclaving. Routinely, 25 mL of molten media was allocated in to culture tubes (25 mm × 150 mm), tightly plugged with plastic caps, and sterilized at 121 °C (15 psi) for 15 min. Averages of 40–50 anthers were inoculated in each test tube after cutting the base of the florets attached to the panicle and tapped the florets on top of the tubes for uniform distribution in media. The cultured anthers were incubated in dark at 25 ± 2 °C during the period of callus induction. Anther response was assessed after three weeks and every week thereafter. Further, calli (1–2 mm) derived from microspores of anthers were transferred to two different semi-solid MS media along with combinations of growth regulators BAP (1.5–2.0 mg/L), Kn (0.5–1.0 mg/L) and NAA (0.5 mg/L) with sucrose (30 g/L) for shoot regeneration (Table 4). The cultures were incubated at temperature of 25 ± 2 °C with 3000 lux illumination (cool, white fluorescent lamps) under 16 h photoperiod. The green plantlets when reached a size of around 5–6 cm in length, were transferred to MS media supplemented with NAA (2.0 mg/L), Kn (0.5 mg/L) and sucrose (50 g/L) for root induction.

### 4.3. Acclimatization

The resulting regenerants with well-formed roots were removed from the culture tube and kept in tap water for 3-d at 27 ± 2 °C along with 50–70% relative humidity for acclimatization. The acclimatized plantlets were transplanted onto 12” pots and placed in net house for which necessary management was taken care for proper growth and development of plants.

### 4.4. Ploidy-Status Assessment in Regenerants

The variation in ploidy level of the regenerants was assessed by evaluating agro-morphological characters such as plant height and spikelet fertility when the plants attained full maturity. Plants showing normal morphological features with fertility were considered diploids. Tall/gigantic growth with thick dark green leaves and less than 1% of spikelet fertility were the morphological indicator to identify tetraploids or polyploids. Haploid plants are supposed to be small and sterile. Simultaneously, the mixploids were identified by visualizing the grain characters in individual tillers. Since morphological characterization cannot discriminate DHs from fertile diploids, STMS (sequence-tagged microsatellite sites) markers were used for identification of true DHs.

### 4.5. STMS Marker Analysis

A total of 650 markers [5] were screened, out of which 38 marker pairs showed heterozygosity with the donor and were used to distinguish DHs from all the developed diploids. Genomic DNA from young leaves were isolated individually from all 324 fertile diploids by a modified cetyltrimethyl ammonium bromide (CTAB) method [18]. Gene amplification reactions were performed with total volume of 10 µL each containing 1× PCR buffer (Kapa Taq PCR Kit, Sigma-Aldrich, St. Louis, MO, US) (75 mM Tris HCl; pH 9.0), 50 mM KCl, 20 mM (NH_4_)_2_SO_4_, 2 mM MgCl_2_, 0.25 mM deoxy nucleotide triphosphates (dNTPs) (Axygen, New Delhi, India), 10 pmol of each of the primers, 1.0 unit of Taq DNA polymerase (Kapa Taq PCR Kit, Sigma-Aldrich, St. Louis, MO, USA), and 25 ng of template DNA. The amplification reaction was carried out in a DNA Thermal Cycler (Eppendorf AG, Hamburg, Germany) programmed for 30 cycles: initial denaturation cycle of 4 min at 94 °C followed by 30 cycles each of 30 s denaturation at 94 °C, 45 s annealing at 55 °C and 1 min extension at 72°C. The final elongation cycle of 10 min at 72 °C marks the completion of polymerization reaction. After completion of the polymerase chain reaction (PCR), 2.5 µL of 6× loading dye (Axygen, New Delhi, India) was added to the amplified products and were electrophorized in a 2.5% (*w/v*) agarose (Genei, Bengaluru, India) gel with 1× Tris-borate ethylene diamine tetraacetic acid (TBE buffer), stained with ethidium bromide and documented by a gel documentation system (Syngene, Cambridge, UK). The size of amplified products was estimated by comparing with standard 100 bp DNA ladder (Genei, Bengaluru, India). All the reactions were repeated twice.

### 4.6. Observations and Statistical Analysis

Each treatment had 20 cultures (each culture containing 40–50 anthers) for callus induction and 15 cultures (each culture containing 4–5 calli) for shoot regeneration; each experiment was repeated twice in two consecutive seasons i.e., kharif (2016–2017) and rabi (2017–2018). The number of calli and green/albino shoots was recorded. The effect of each treatment was evaluated as against of their respective controls. The following formulae were employed:

$$\mathrm{Callus}\;\mathrm{response}\;(\%)\;=\;\frac{No.\;of\;Anthers\;forming\;callus}{Total\;no.\;of\;anthers\;cultures}\times 100$$

$$\mathrm{Plant}\;\mathrm{Regeneration}\;(\%)\;=\;\frac{No.\;of\;calli\;producing\;green\;plants}{Total\;no.\;\;of\;calli\;transferred}\times 100$$

Statistical analysis of each parameter related to androgenesis were analyzed as completely randomized design (CRD) by using WASP1.0 software (ICAR-CCARI, Goa). All data were subjected to analysis of variance (ANOVA). The means were separated according to Duncan’s multiple range test (DMRT).

## 5. Conclusions

In this study, an efficient androgenic protocol was established in *indica* rice hybrid 27P63, by manipulation of physical and chemical factors. In addition, standardization of androgenic response in 27P63 ratooned plants, provided an insight for variable responses between rice hybrid and ratooned plant, giving an idea for optimization of callusing and green shoot regeneration by simply manipulating the PGRs in the media. Besides, varying the cut distance of the main crop could address the reduction of androgenic response in the ratooned plant vis-a-vis the main plant, which could help in establishment of an efficient androgenic protocol for ratooned plants.

## Figures and Tables

**Table 1:** Response of PGRs in two basal media (N6 and MS) with duration of cold pre-treatment on callus induction in non-ratooned and ratooned anthers of 27P63.

Explant Source	Days of Pre-Treatment	Callus Response in “N6” Media *			Callus Response in “MS” Media *		
		N-1	N-2	N-3	M-1	M-2	M-3
Non ratooned	2	21.66 ± 0.75 a	9.00 ± 0.17 a	2.26 ± 0.15 a	13.66 ± 0.05 a	9.66 ± 0.15 a	7.10 ± 0.43 a
	7	14.33 ± 0.35 b	3.96 ± 0.35 b	1.50 ± 0.43 b	8.53 ± 0.35 b	5.00 ± 0.52 b	3.73 ± 0.32 b
	8	10.00 ± 0.26 d	1.93 ± 0.32 d	0.86 ± 0.30 d	5.83 ± 0.20 c	3.00 ± 0.30 c	2.70 ± 0.45 c
Ratooned	2	11.66 ± 0.30 c	2.66 ± 0.15 c	1.20 ± 0.20 c	2.66 ± 0.40 d	0.63 ± 0.15 d	0.33 ± 0.15 d
	7	6.67 ± 0.32 e	0.66 ± 0.15 e	0.30 ± 0.26 e	1.33 ± 0.15 e	0.10 ± 0.10 e	0
	8	2.66 ± 0.20 f	0.36 ± 0.35 f	0	0.66 ± 0.41 f	0	0

Means sharing the same letter in a column were not significantly different in Duncan’s multiple comparison range test (*p* < 0.05). * 20 replicates per treatment; repeated twice.

**Table 2:** Response of N6, MS media grown calli for shoot regeneration in MS media with growth regulators derived from anthers of non-ratooned and ratooned of 27P63.

Source of Explant	Calli Induction Media	Green Shoot Regeneration (%) in MS Media + Growth Regulators *		Source of Explant	Calli Induction Media	Green Shoot Regeneration (%) in MS Media + Growth Regulators *	
		MS1	MS2			MS1	MS2
Non-ratooned	N1	76.00 ± 0.30 a	27.66 ± 0.15 a	Ratooned	N1	38.86 ± 0.26 a	19.96 ± 0.25 a
	N2	37.66 ± 0.15 b	14.33 ± 0.14 d		N2	18.16 ± 0.14 b	13.40 ± 0.17 b
	N3	29.33 ± 0.35 c	11.66 ± 0.25 e		N3	13.16 ± 0.13 c	13.00 ± 0.33 bc
	M1	22.33 ± 0.37 d	20.13 ± 0.35 b		M1	10.63 ± 0.30 e	12.53 ± 0.35 d
	M2	15.33 ± 0.25 e	16.66 ± 0.05 c		M2	12.53 ± 0.25 d	11.30 ± 0.37 e
	M3	13.33 ± 0.30 f	0		M3	0	0

Means sharing the same letter in a column were not significantly different in Duncan’s multiple comparison range test (*p* < 0.05). * 15 replicates per treatment; repeated twice.

**Table 3:** Two different basal media MS and N6 with three different combinations and concentrations of growth regulators for callus induction.

Basal Media + Growth Regulators	Media Combinations	2,4-D (mg/L)	BAP (mg/L)	Kn (mg/L)
N6 media	N1	2.0	0.5	--
	N2	1.5	--	0.1
	N3	2.5	1.0	--
MS media	M1	2.0	0.5	0.1
	M2	2.0	--	0.1
	M3	2.5	1.0	--

**Table 4:** MS media supplemented with combinations of PGR for regeneration.

Semi-Solid MS Media	BAP (mg/L)	Kn (mg/L)	NAA (mg/L)
MS1	1.5	0.5	0.5
MS2	2.0	1.0	0.5
